# Short Report: Using Targeted Urine Metabolomics to Distinguish Between Manganese Exposed and Unexposed Workers in a Small Occupational Cohort

**DOI:** 10.3389/fpubh.2021.666787

**Published:** 2021-05-20

**Authors:** Kayla A. Carter, Christopher D. Simpson, Daniel Raftery, Marissa G. Baker

**Affiliations:** ^1^Department of Epidemiology, University of Washington, Seattle, WA, United States; ^2^Department of Environmental and Occupational Health Sciences, University of Washington, Seattle, WA, United States; ^3^Northwest Metabolomics Research Center, University of Washington, Seattle, WA, United States

**Keywords:** occupational health, exposure assessment, targeted metabolomics, manganese exposure, biomonitoring

## Abstract

**Objectives:** Despite the widespread use of manganese (Mn) in industrial settings and its association with adverse neurological outcomes, a validated and reliable biomarker for Mn exposure is still elusive. Here, we utilize targeted metabolomics to investigate metabolic differences between Mn-exposed and -unexposed workers, which could inform a putative biomarker for Mn and lead to increased understanding of Mn toxicity.

**Methods:** End of shift spot urine samples collected from Mn exposed (*n* = 17) and unexposed (*n* = 15) workers underwent a targeted assay of 362 metabolites using LC-MS/MS; 224 were quantified and retained for analysis. Differences in metabolite abundances between exposed and unexposed workers were tested with a Benjamini-Hochberg adjusted Wilcoxon Rank-Sum test. We explored perturbed pathways related to exposure using a pathway analysis.

**Results:** Seven metabolites were significantly differentially abundant between exposed and unexposed workers (FDR ≤ 0.1), including n-isobutyrylglycine, cholic acid, anserine, beta-alanine, methionine, n-isovalerylglycine, and threonine. Three pathways were significantly perturbed in exposed workers and had an impact score >0.5: beta-alanine metabolism, histidine metabolism, and glycine, serine, and threonine metabolism.

**Conclusion:** This is one of few studies utilizing targeted metabolomics to explore differences between Mn-exposed and -unexposed workers. Metabolite and pathway analysis showed amino acid metabolism was perturbed in these Mn-exposed workers. Amino acids have also been shown to be perturbed in other occupational cohorts exposed to Mn. Additional research is needed to characterize the biological importance of amino acids in the Mn exposure-disease continuum, and to determine how to appropriately utilize and interpret metabolomics data collected from occupational cohorts.

## Introduction

Manganese (Mn) is a known neurotoxicant associated with a range of motor (1–3) and cognitive (4–6) health outcomes. Elevated exposure to Mn occurs most frequently in metal-working occupational settings, such as among solderers, welders, brazers, and foundry workers. While environmental Mn exposures are typically lower than in occupational settings, elevated environmental exposures can occur in proximity to Mn-utilizing industrial facilities or busy roadways, putting more people at risk for health outcomes related to Mn (7–9).

The gold-standard for measuring airborne Mn exposure remains filter-based personal air sampling, though a variety of exposure biomarkers have been explored in both occupational and environmental cohorts (10–14). However, notable limitations to using common biologic matrices, such as urine, blood, and plasma, to assess Mn exposure have been discussed in the literature (15). Magnetic resonance imaging (MRI) and positron emission tomography (PET) have been found to be promising assessment methods across a range of exposures; however, contraindications to these procedures and the cost and specialized equipment required can reduce their utility for routine use (16–18).

Given the limitations of common Mn exposure assessment methods, there is a public health interest in investigating readily accessible biomarkers related to Mn exposure, such as urinary metabolites that distinguish between exposed and unexposed persons. These small molecule metabolites could serve as putative biomarkers of Mn exposure and help elucidate the biological processes that Mn may perturb either simultaneously with or prior to exerting neurotoxicity. Metabolomics is the study of these small molecules (<1,500 Daltons) that are important in metabolic processes. Previously, we have utilized global metabolomics profiling to investigate metabolites that differ between exposed and unexposed workers in the Puget Sound region, whose data are also used in this manuscript (19). In our previous work, we found nine metabolites to be significantly differentially abundant between exposed and unexposed workers [false discovery rate (FDR) <0.1], and most of these metabolites also exhibited an exposure-response relationship when stratifying workers by no exposure, low exposure, and high exposure. However, when investigating these nine metabolites in a different occupationally exposed cohort of welders, these nine metabolites were no more predictive of exposure status than by chance alone (20). As this previous work utilized global metabolomics methods, the identity of the nine metabolites were not known, making it challenging to infer biologic relevance as to why they may not have replicated in another occupational cohort. To improve on this limitation, targeted metabolomics, where the identity of the metabolite is known, was utilized in the Puget Sound cohort to investigate not only metabolite differences between groups defined by exposure, but also potential pathway perturbations related to Mn exposure in these workers, which could inform how Mn exerts toxicity in exposed individuals.

## Methods

### Study Population and Samples Collected

Foundry workers at a Mn-steel foundry and crane operators or truck drivers at a scrap metal recycling yard were organized into a meeting by their site health and safety officer. Here, workers were given an overview of the study by the study team, given a chance to ask questions, and interested participants were enrolled in our study after giving written, informed consent. All study protocols were approved by the University of Washington Institutional Review Board (IRB number 47550). A total of 20 Mn-exposed foundry workers and 17 Mn-unexposed crane operators and truck drivers were recruited into our study. Both workplaces are located in the Puget Sound region of Washington state. The characteristics of the cohort and details on the study design have been previously described by Baker et al. (19). In October 2014, a full-shift personal air sample was collected from these workers to ensure there was not exposure misclassification between the exposed and unexposed groups. Airborne Mn exposure was assessed using Institute of Medicine (IOM) inhalable dust samplers, which were analyzed for the inhalable Mn fraction according to the UK Health and Safety Executive's Methods for the Determination of Hazardous Substances 14/4 (21). The mean 8-h time weighted average (TWA) Mn exposure in the foundry workers was 365 μg/m^3^ [standard deviation (SD): 300, range: 98.5, 1,243] whereas the mean Mn exposure for the crane operators/truck drivers was 9.2 μg/m^3^ (SD: 36.5, range: 0.02, 150.8), confirming the expected Mn exposure difference between the exposed and unexposed workers. The mean 8-h TWA Mn exposure in the foundry workers exceeded the American Conference of Governmental Industrial Hygienist's (ACGIH) threshold limit value (TLV) of 100 μg/ m^3^, while the mean exposure for the crane operators/truck drivers was well below this limit.

An end-of shift spot urine sample was collected from 17 Mn-exposed foundry workers and 15 unexposed crane operators or truck drivers in January 2015. Clean catch urine samples were collected in 125 mL wide mouth low-density polypropylene bottles (Nalgene). Urine samples were aliquoted on site into 2 mL Safe-Lock Eppendorf tubes, and immediately stored on dry ice for transport to University of Washington (UW) in Seattle, where they were stored at −80°C awaiting metabolomics analysis. Participants completed a short exposure questionnaire when their urine was collected in January 2015. The goal of this questionnaire was to confirm that current job duties were similar to job duties in October 2014 when personal airborne exposure had been assessed.

### Targeted Metabolomics Analysis

A targeted assay of 362 aqueous metabolites was undertaken via liquid chromatography-tandem mass spectrometry (LC-MS/MS) on the urine samples. Sample preparation and analysis was completed by the Northwest Metabolomics Research Center at UW. Frozen urine samples were thawed on wet ice and vortexed. Next, 100 μL of urine were mixed with methanol in a 2:1 ratio to precipitate any residual protein, and a solution containing 32 isotope-labeled internal standards was added to the urine-methanol mixture to monitor sample preparation and quantitate metabolites. After drying the samples using a Vacufuge (Eppendorf), samples were reconstituted in mobile phase B solvent (see below) and diluted by 5:1 prior to LC-MS/MS analysis.

These Puget Sound samples were randomized with urine samples from a separate study undergoing the same metabolomics analysis in a three batch run. A pooled quality control (QC) sample was constituted using small aliquots from the study samples, and it was run once for every ten study samples for a total of 12 pooled QC samples. In addition, a pooled instrument control sample (serum) was also run once every 10 study samples, along with blank samples. Samples were injected into the chromatography system (CDC autosampler and Shimadzu Nexera LC-20 pumps) consisting of a dual injection valve setup allowing injections onto two different LC columns with each column dedicated to an ESI polarity. For positive mode ionization, 5 μL were injected on to the column, and 10 μL on to the column for negative mode. Both columns were Waters XBridge BEH amide columns (2.1 × 150 mm) from the same production lot. The autosampler was maintained at 4°C and the column oven was set to 40°C. Mobile phase A was 10 mM ammonium acetate in 95% water, 3% acetonitrile, 2% methanol, and 0.2% acetic acid, and Mobile phase B was 10 mM ammonium acetate in 93% acetonitrile, 5% water, 2% methanol, and 0.2% acetic acid. The 0.3 mL/min solvent gradient was as follows: 0–1.5 min 95% mobile phase B, 1.5–6 min 95% to >70% B, 6–10 min 70% B, 10–12 min 70–45% B, 12–14 min 45% B, 14–15 min 45% to >95% B, 15–18 min 95% B. After completion of the 18 min gradient, injection on the other column was initiated and the inactive column was allowed to equilibrate at the starting gradient conditions. A set of QC injections for both instrument and sample QC were run at the beginning and end of the sample batch, as well as every 10 study samples. Blank samples were run periodically as well to monitor carryover.

The MS data were integrated using SCIEX MultiQuant 3.0.2 or Sciex-OS v1.5 software. Peaks were selected based on peak shape, a signal-to-noise ratio of >10, and retention times consistent with previously run standards and sample sets. The median metabolite coefficient of variation (CV) in the pooled QC samples over the course of the run was 7.9%; the median metabolite CV for the pooled laboratory standard QC samples over the course of the run was 7.8%.

### Statistical Analysis

Metabolites which were not present above the limit of detection in at least 50% of the samples were removed, resulting in a total of 224 metabolites (62%) included in our sample set. Missing values were replaced with 1 × 10^−6^ prior to normalization and log_10_-transformation. In order to account for systematic errors resulting from instrument drift and differences in urine dilution and hydration between the participants, samples were normalized in Metaboanalyst 4.0 by the set of pooled QC samples (22), a step undertaken for each individual metabolite measured. This normalization step is important for reducing systematic variation and allowing true biological variation between the samples to be revealed (23). For this type of normalization, pooled probabilistic quotient normalization (PQN) is used, which looks at the distribution of metabolites across the pooled QC samples and adjusts the participant samples based on the QC samples, therefore relying on reference samples instead of the study samples themselves (23). This method also adjusts for differences in dilution by determining a probabilistic dilution factor for each sample, based on the differences between each sample and the pooled QC samples. After applying this normalization to the pooled QC samples, the median metabolite CV over the course of the run decreased to 4.1%.

Prior to statistical analyses, data were log_10_-transformed. As data were assumed to be non-parametric, relative abundances of all 224 metabolites were compared between the exposed and unexposed workers using a Wilcoxon rank sum test. *P*-values were adjusted using the Benjamini-Hochberg method to control false discovery rates. In order to find a larger potential set of metabolites that could distinguish between exposure groups, a false discovery rate (FDR) ≤ 0.1 was considered significant.

For the metabolites found to be significantly differentially abundant between the exposed and unexposed groups, the exposure-response relationship was explored by using box-plots to visualize relative abundances in Mn exposed and unexposed groups. Data analysis was completed in R (version 3.6.1) and R Studio (version 1.2).

To identify pathways that may have been perturbed between individuals exposed and unexposed to Mn, a pathways analysis of the 224 metabolites was carried out using MetaboAnalyst 4.0 (22). This MetaboAnalyst module combines an enrichment analysis, which calculates whether groupings of metabolites in the same metabolic pathway differ significantly between exposure groups, and a pathway topology analysis, which assigns an impact score to each pathway. A higher impact score is indicative of not only more significant perturbations in the pathway, but also biologically meaningful changes in the measured metabolites (24).

To undertake the pathways analysis, the 224 metabolites identified from the targeted assay were matched to their Human Metabolome Database identifier for upload to MetaboAnalyst 4.0, where their relative abundances by exposure group were compared to the KEGG (Kyoto Encyclopedia of Genes and Genomes) metabolites pathway library for *Homo sapiens*. The Global Test option in MetaboAnalyst 4.0 was used to evaluate relative abundance differences among groups of metabolites in the same metabolic pathway, with these differences being used to calculate Benjamini-Hochberg FDRs between the exposed and unexposed groups. The betweenness centrality option (shortest path between nodes) was used to calculate metabolite importance (25, 26). With this method, the location of the metabolite in the pathway is considered, so when perturbed metabolites are central to the pathway and operate near each other or in succession to each other, it is assumed the pathway could be more impacted. If perturbed metabolites are only marginal to the pathway or relatively isolated compared to other perturbed metabolites in the pathway, then that pathway receives a lower impact score (27).

## Results

Table 1 shows participant demographics for the Mn-exposed and -unexposed workers included in this study. While both exposed and unexposed workers were predominantly White, there were more Hispanic workers in the Mn-exposed group. The Mn-exposed group was also slightly younger than the Mn-unexposed group. Nearly half of the Mn-unexposed workers were working on a night shift, whereas the Mn-exposed workers were predominantly first shift workers. No Mn-unexposed workers wore a respirator, but 10 of the Mn-exposed workers self-reported wearing an N95 or dust mask at least some of the time on the day their urine sample was collected. The lack of a formal respiratory protection program at the foundry and the observed poor respiratory hygiene allowed us to infer any mask use would have a limited effect on the received Mn dose.

**Table 1:** Participant demographics.

	**Mn-exposed workers**	**Mn-unexposed workers**
	**(*n* = 17)**	**(*n* = 15)**
	**Mean ± SD (range)**	**Mean ± SD (range)**
Age (at time of sample)	43.1 ± 12.0 (25, 66)	49.3 ± 10.1 (26, 60)
	***n*** **(%)**	***n*** **(%)**
**Ethnicity**		
Hispanic	9 (53)	4 (27)
Non-Hispanic	8 (47)	11 (73)
**Race**		
White	13 (76)	13 (87)
Non-White	4 (24)	2 (13)
**Respirator**		
Yes	10 (59)	0 (0)
No	7 (41)	15 (100)
**Smoker**		
Current	2 (13)	3 (18)
Previous	7 (47)	9 (53)
Never	6 (40)	5 (29)
**Shift**		
First	15 (88)	8 (53)
Second	2 (12)	7 (47)

**Figure 1:**
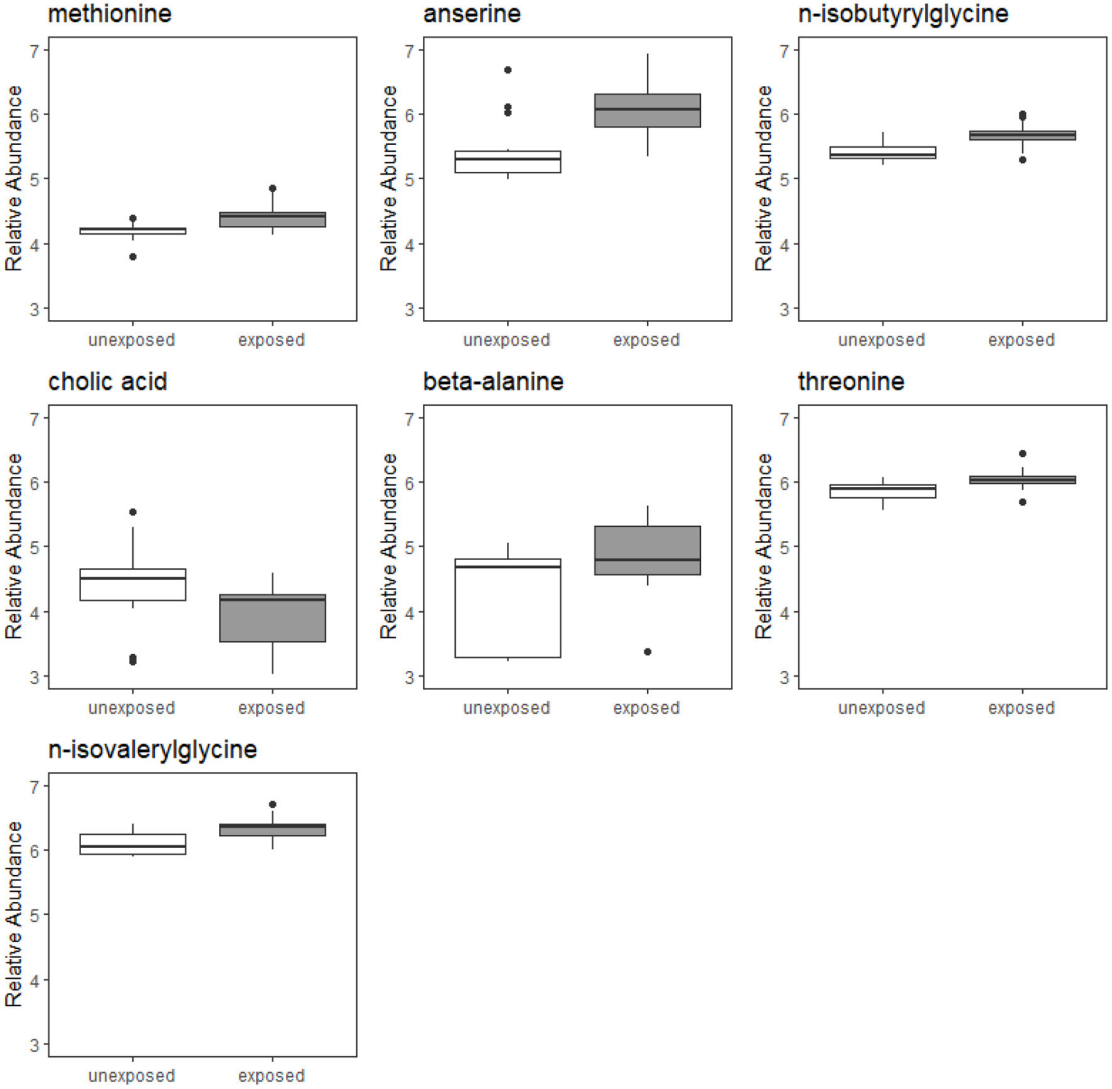
Boxplots of relative abundances of seven metabolites found to significantly differentially abundant between Mn-exposed and -unexposed workers. For each exposure group, the middle line that divides the box into two parts represents the median value while the top and bottom lines of the box represent the 75th and 25th percentiles, respectively. The box represents the interquartile range (IQR) of scores for the group. The whiskers are extended to all values that are no >1.5 × IQR from the edge of the box.

Of the 224 metabolites identified from the targeted assay, seven were found to be significantly differentially abundant between groups defined by Mn exposure at an FDR ≤ 0.1: n-isobutyrylglycine, cholic acid, anserine, beta-alanine, methionine, n-isovalerylglycine, and threonine. These seven metabolites are outlined in Table 2, and boxplots showing their abundances in the exposure groups are shown in Figure 1. Table 2 also provides information on their source, chemical class, and biological role in human metabolism. Results from Wilcoxon rank sum test for all 224 metabolites (including their adjusted *P*-values) are included in the Supplementary Material.

**Table 2:** Normalized, log_10_-transformed relative abundance of the seven metabolites found to be significantly differentially abundant (FDR ≤ 0.10) between Mn-exposed and -unexposed participants, and their biological details.

	**Mn-exposed (*****n*** **=** **17)**			**Mn-unexposed (*****n*** **=** **15)**							
**Metabolite**	**Mean (SD)**	**CV (%)**	Detected %^*^	**Mean (SD)**	**CV (%)**	Detected %^*^	FDR^**^	**ESI**	**Chemical class**	**Source**	**Biological role**
n-Isobutyrylglycine (28)	5.68 (0.19)	3	100	5.40 (0.15)	3	100	0.02	Negative	Acylglycine	Endogenous	Urinary metabolite of fatty acids
Cholic acid (29)	3.43 (0.77)	22	41	4.41 (0.62)	14	87	0.03	Negative	Bile acid	Endogenous	Facilitates fat absorption and cholesterol excretion
Anserine (30)	6.25 (0.69)	0.1	100	5.42 (0.48)	9	100	0.03	Negative	Dipeptide	Endogenous	Biochemical buffer, chelator, antioxidant, and anti-glycation agent
Beta-alanine (31)	4.50 (0.98)	22	76	3.26 (0.84)	26	20	0.03	Positive	Beta amino acid	Endogenous	Aids in production of carnosine, which plays a role in muscle endurance
Methionine (32)	4.42 (0.19)	4	100	4.19 (0.15)	4	100	0.03	Positive	Amino acid	Exogenous	Important in growth of blood vessels, protein biosynthesis
n-Isovalerylglycine (33, 34)	6.33 (0.20)	3	100	6.10 (0.19)	3	100	0.08	Negative	Acylglycine	Endogenous	Urinary metabolite of fatty acids
Threonine (35)	6.03 (0.16)	3	100	5.86 (0.13)	2	100	0.10	Positive	Amino acid	Exogenous	Protein biosynthesis, deficiency related to neurological dysfunction

* *Detected % refers to the number of samples in which this metabolite was detected*.

** *Benjamini-Hochberg adjusted p-value between Mn-unexposed (n = 15) and Mn-exposed (n = 17) participants, from Wilcoxon rank-sum test*.

*FDR, false discovery rate; SD, standard deviation; CV, coefficient of variation; ESI, electrospray ionization mode*.

A pathways analysis of 80 *Homo sapiens* metabolic pathways was undertaken with the normalized data to determine if different metabolic pathways were perturbed between the Mn-unexposed and -exposed groups. Twenty-three pathways were identified that included at least four of the 224 metabolites, which is considered the minimum number for meaningful pathway analysis (36, 37). Of these 23 pathways, seven had a FDR < 0.1, indicating a significant perturbation in the pathway between exposed and unexposed workers: beta-alanine metabolism, propanoate metabolism, pyrimidine metabolism, pantothenate and CoA biosynthesis, primary bile acid biosynthesis, histidine metabolism, and glycine, serine, and threonine metabolism. Of these significantly perturbed pathways, three had an impact score >0.5, indicating the perturbed metabolites in that pathway were at least moderately central to the pathway and operate near or in succession to each other: beta-alanine metabolism, histidine metabolism, and glycine, serine, and threonine metabolism. Table 3 outlines the pathways that were found to be significantly perturbed between exposed and unexposed workers, including the impact scores for each of these pathways.

**Table 3:** Pathway enrichment analysis.

**Pathway**	Compounds in pathway^*^	Compounds in data^*^	FDR^**^	**Impact**
Beta-alanine metabolism	21	7	0.003	0.56
Propanoate metabolism	23	5	0.01	0.04
Pyrimidine metabolism	39	8	0.01	0.19
Pantothenate and CoA biosynthesis	19	6	0.01	0.06
Primary bile acid biosynthesis	46	6	0.01	0.04
Histidine metabolism	16	7	0.05	0.64
Glycine, serine, and threonine metabolism	33	16	0.08	0.66

* *Compoundsin pathway refers to the total number of compounds operating in that pathway, including compounds that were not investigated in our dataset; Compounds in data refers to the number of compounds in the pathway that were in our data set of 224 metabolites*.

** *Benjamini-Hochberg adjusted p-value characterizing the differences in the pathways between Mn unexposed (n = 15) and Mn exposed (n = 17) participants*.

*FDR, false discovery rate*.

## Discussion

Here, differences in 224 metabolites measured using a targeted metabolomics LC-MS/MS platform were explored between Mn-exposed and -unexposed workers. Seven metabolites were found to be significantly differentially abundant between exposure groups. When investigating which pathways were perturbed in exposed workers as compared to unexposed workers, pathways related to amino acid metabolism (beta-alanine metabolism, histidine metabolism, and glycine, serine, and threonine metabolism) were significantly perturbed in the Mn-exposed group and had the highest impact scores.

While differences in metabolite abundance between Mn-exposed and -unexposed workers have not previously been investigated using a targeted metabolomics platform, others have examined differences in metabolomics profiles between workers exposed and unexposed to welding fume, which typically includes high levels of Mn. In a study of 52 boilermakers, Shen et al. (38) utilized an untargeted metabolomics approach to look at changes in plasma collected pre- and post-welding shift. These untargeted data were generated using a mass spectrometry platform. The authors found that the metabolic changes over the work shift were related to changes in lipid pathways and amino acid utilization, both of which are associated with inflammation. Wang et al. (34) compared the urine metabolomics profile of 10 welders and 6 office workers using untargeted data generated from a nuclear magnetic resonance (NMR) platform. After identifying the NMR bins found to be significantly different between groups, the authors found higher levels of several amino acids, creatinine, and acetone among welders, and lower levels of creatine. The authors hypothesized many compounds found to be higher in welders are important in modulating inflammatory and oxidative tissue injury processes. Notably, to control for some potential confounding, Wang et al. only included participants who did not smoke cigarettes or drink alcohol and took urine samples after overnight fasting, whereas similar steps were not taken in the study presented here.

Despite representing three different cohorts exposed to Mn-containing fumes and metabolomics data generated from three different analysis platforms, Shen et al., Wang et al., and the work presented here all found amino acid perturbations in groups exposed to Mn-containing fumes. Further research is needed to understand the potential importance of amino acids in the Mn exposure-disease continuum, and whether elevated levels of particular amino acids are consistently related to exposure to Mn.

For exposure studies utilizing metabolomics, it can be challenging to determine if the differences between groups are truly due to the measured exposure, or if unmeasured co-exposures in the workplace may be driving the observed differences. Foundry work could have a number of co-exposures that differ from those encountered by crane operators/truck drivers at a metal recycling center. These include substantial exposure to silica and carbon monoxide, in addition to polycyclic aromatic hydrocarbons (PAHSs), phenol, formaldehyde, isocyanates, and amines among foundry workers. Foundry workers could also have exposures to other metals such as chromium, nickel, and iron. In this study, co-exposures were not assessed due to operational constraints. Metabolites are also subject to a variety of internal and external cues, and bodily concentrations dynamically change throughout the day due to circadian rhythms, activities the person is performing, food the person is eating, and other constantly occurring internal biological processes. For example, two of these seven metabolites of interest to this study are essential amino acids, which likely differ between the groups due to differences in diet as that is their predominant source.

Here, urine samples were collected from each subject at the end of their work shift, but this would represent different clock times for workers on a first shift and second shift, which could influence metabolite levels. However, when stratifying the seven metabolites presented in Table 2 by shift for unexposed workers, no differences in distributions were seen, though power to detect differences was limited. While it is impossible to control all sources of within- and between-person variability in an occupational setting, care should be taken to ensure samples are taken at the same time of day. Appropriately-timed repeat samples can also be informative for understanding the variability in changes to metabolites related to exposure.

Similarly, co-variates were not collected on biological and behavioral characteristics that can influence metabolomics, including body mass index (BMI), pre-existing conditions, dietary habits, or use of pharmaceutical agents. Unfortunately, the lack of repeat measures, lack of information on co-exposures, and lack of co-variates collected must be acknowledged as a major limitation in this study and something that should be accounted for in future occupational metabolomics studies.

Additionally, we did not undertake any validation of study findings to see if the metabolites found to be significantly differentially abundant in these exposed workers remained so in other Mn-exposed workers, or in a testing set of samples from this cohort. This would be an important step to increase the external validity of this study, and to further confirm the biological relevance of the findings. Future occupational metabolomics studies should strive to enroll sufficient participants to split their data into separate training and testing sets, or utilized data from an external dataset for testing and validation purposes. The sample size of this study, which was originally conceived of as a pilot study, was a major contributor to the lack of power to detect differences between the 224 compounds investigated here.

The potential value of targeted metabolomics for occupational exposure studies must also be noted. Targeted metabolomics allows the occupational health researcher to investigate a range of known metabolites that may relate to different exposures the worker has experienced, both at work and in other environments. This makes metabolomics an important tool for characterizing the exposome, which refers to the totality of exposures that someone has encountered throughout their lifetime (39). Occupational settings are a particularly valuable place to develop metabolomics methods for exposure assessment, given the higher exposures typically experienced in workplace environments and the prevalence of otherwise uncommon exposures in workplace environments. With work schedules following consistent patterns, it can also help with ensuring consistency in sample collection.

In conclusion, this work continued to explore the utility of metabolomics for distinguishing between groups defined by occupational exposures. Findings from this study were consistent with other studies of workers exposed to Mn-containing fumes, showing perturbations of amino acids and amino acid pathways. While further study is warranted to explore the potential role of amino acids in Mn toxicity, we hope that the work presented here encourages others to integrate targeted metabolomics into their human exposure studies, in order to continue to expand the use of this promising technique as a means of hypothesis generation and biomarker discovery in occupational health and exposome studies. We also hope that the limitations we outlined here will ensure that subsequent occupational health researchers can collect more rigorous co-variates with their biosamples to better inform results from planned or subsequent metabolomics analyses.

## Data Availability Statement

The datasets generated for this study can be found in online repositories. The names of the repository/repositories and accession number(s) can be found below: github.com/bakermarissa/targeted_mn_metabolomics.

## Ethics Statement

The studies involving human participants were reviewed and approved by University of Washington IRB. The patients/participants provided their written informed consent to participate in this study.

## Author Contributions

MGB and CDS conceived of and designed the original research study. MGB completed all data collection and secured funding for analyses presented here. DR oversaw all laboratory analyses. MGB, KAC, and DR undertook data analysis and interpretation. KAC and MGB drafted the article. KAC, MGB, CDS, and DR undertook critical revision of article. All authors gave final approval of the version to be published and as such all agree to be held accountable for the work.

## Conflict of Interest

The authors declare that the research was conducted in the absence of any commercial or financial relationships that could be construed as a potential conflict of interest.
